# CHARON: estimating the drift time and the number of individuals in environmental DNA with diploid individuals

**DOI:** 10.1093/g3journal/jkag099

**Published:** 2026-05-12

**Authors:** Jan van Waaij, Mads Vodder Hartmann, Peter Wad Sackett, Gabriel Renaud

**Affiliations:** Department of Health Technology, Section for Bioinformatics, Technical University of Denmark, Ørsteds Plads, Building 345C, Kongens Lyngby DK-2800, Denmark; Department of Health Technology, Section for Bioinformatics, Technical University of Denmark, Ørsteds Plads, Building 345C, Kongens Lyngby DK-2800, Denmark; Department of Health Technology, Section for Bioinformatics, Technical University of Denmark, Ørsteds Plads, Building 345C, Kongens Lyngby DK-2800, Denmark; Department of Health Technology, Section for Bioinformatics, Technical University of Denmark, Ørsteds Plads, Building 345C, Kongens Lyngby DK-2800, Denmark; Department of Computer Science and Software Engineering, Université Laval, Québec City, Québec, Canada

**Keywords:** drift estimation, eDNA, number of individuals

## Abstract

Environmental DNA (eDNA) offers a promising avenue for reconstructing the genetic diversity and demographic history of ancient populations. However, the analysis of eDNA from humans or forensic DNA, presents significant challenges, including low coverage, DNA degradation, and the uncertainty of the number of individuals contributing to a sample. This study introduces CHARON, a novel statistical method for jointly estimating the number of individuals and drift times between a human eDNA or forensic sample and a given population with known allele frequencies. We validate our method through simulations and synthetic empirical data and show that we can reliably estimate the number of individuals up to 8 at a coverage between 2X and 4X. Our method can also pinpoint the most likely population of origin for eDNA or forensic samples. This work provides a tool for the application of human eDNA in evolutionary and forensic studies and an implementation is available here: https://github.com/Jan-van-Waaij/Charon.

## Introduction

Environmental DNA (eDNA) has rapidly evolved into a transformative tool for ecological monitoring, enabling the detection of species in various environments without the need for physical capture or direct observation (Beng and Corlett 2020). By collecting and analyzing DNA fragments shed by organisms into their surroundings—whether it be in water, soil, or air—researchers can identify the species present in an area, offering a noninvasive means of studying biodiversity (Cristescu and Hebert 2018; Deiner et al. 2021). For example, airborne eDNA has been effectively utilized to track seasonal changes in plant communities and to assess the impact of human activities on biodiversity at a landscape scale (Allwood et al. 2020; Johnson et al. 2021). Moreover, the ability to capture eDNA from various environments, including air and water, has opened new avenues for environmental monitoring that can respond rapidly to ecological shifts, such as those induced by climate change or human encroachment (Neves and Zieger 2023; Fantinato et al. 2024).

The applications of eDNA extend beyond ecological studies into the realm of forensic science, where it is being increasingly recognized as a valuable method for the noninvasive collection of human DNA. In forensic contexts, eDNA has demonstrated significant potential in scenarios where traditional DNA sampling methods may be challenging, such as in aquatic environments or across large areas (Williams et al. 2017; Chapman et al. 2023; Fantinato et al. 2024; Lewis et al. 2024; Shahzad et al. 2024). Some studies have indeed shown that eDNA can be used to recover human DNA from water, offering new avenues for locating human remains or tracing human presence in forensic investigations (Dass et al. 2022, 2024). Additionally, the use of airborne eDNA in forensic investigations could provide critical evidence in crime scene analyses, especially in situations where physical evidence is scarce (Primorac and Schanfield 2023; Goray et al. 2024). As these methods continue to be refined, they hold promise for enhancing the capabilities of forensic science, providing innovative and less invasive approaches to evidence collection and analysis (Butler 2015; Wells and Škaro 2023).

An issue facing both the forensics DNA community as well as the ancient DNA community is the presence of multiple individuals in the mix (Prüfer et al. 2010; Orlando et al. 2021). The present-day human individuals involved in either excavation or library preparation can mix their DNA with the DNA from the ancient bones and bespoke computational methods had to be developed to quantify the contamination (Renaud et al. 2015; Peyrégne and Peter 2020). Racimo and Renaud et al. developed a method to jointly estimate present-day contamination as well as demographic parameters (Racimo et al. 2016). This model revolved around computing the posterior probability of the genotype and divergent bases were either due to (i) contamination error, (ii) heterozygous sites, or (iii) sequencing errors. The demographic parameters that were inferred were drift times which provided the prior on the genotype. Later, Schraiber et al. generalize this model to multiple samples (Schraiber 2018).

Inferring drift times between populations is crucial for understanding population structure and history, as it allows us to reconstruct much of this history from the genomes of contemporary individuals. For human eDNA or forensic DNA, drift times to other populations would be informative to provide the ethnicity of the individuals in the mix. Another key piece of information for forensics DNA and human eDNA is the number of individuals that contributed to the blend.

**Fig. 1.**
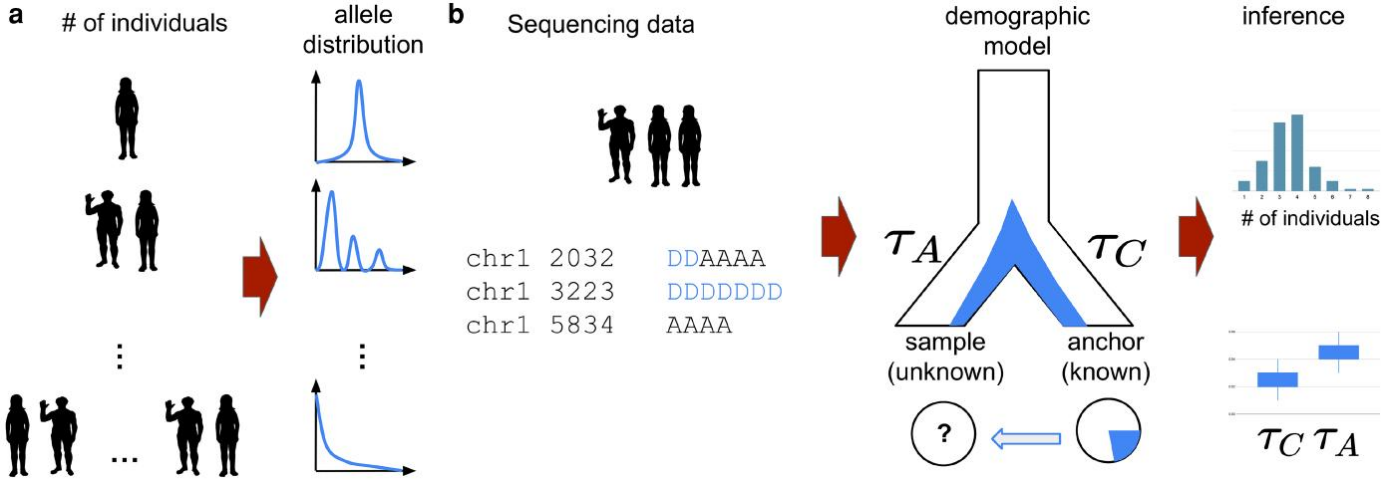
The general workflow for estimating the number of individuals. a) For a single individual with 2 chromosomes, the distribution of the bases around polymorphic sites will follow a binomial distribution around 0.5. For two individuals thus carrying 4 chromosomes, the distribution will be skewed around 0.25, 0.5 and 0.75 when 1 allele is present, 2 and 3, respectively. As the number of individuals increases, this distribution takes the shape of the site frequency spectrum. b) Our method relies on taking base counts from samples of numerous individuals for which we know the allele frequency in an anchor population i.e. a population that is relatively close with well-resolved allele frequencies. Drift parameters are estimated along with an estimate of the number of individuals. A ‘D’ refers to a derived allele and an ‘A’ refers to an ancient allele.

To address the challenges of analyzing eDNA and forensic DNA with multiple contributing individuals, we introduce CHARON, a novel statistical method that jointly estimates both the number of individuals and the drift times between a forensic sample and a modern reference population (see Fig. 1). Our approach leverages allele frequencies from known populations to infer these parameters, providing a more accurate understanding of the genetic makeup of the samples.

We make the following two assumptions about the data: (i) they are all from the same population in the genetics sense and (ii) they have all had more or less the same contribution to the sampled data. By employing simulations and synthetic empirical data, we demonstrate that our method is robust across varying levels of coverage, reliably estimating up to eight individuals with moderate coverage and accurately pinpointing the population of origin. We provide an implementation of our methods written in the Julia programming language here https://github.com/Jan-van-Waaij/Charon.

## Materials and methods

### Test data generation

#### Simulated data

We generated two types of simulated data: (i) a very simple demographics scenario with 2 populations and known drift parameters to determine whether our approach could accurately estimate them and (ii) a more complex demographic model based on human history to see whether our method could pinpoint the population of origin. To simulate genetic data, we used msprime (Kelleher et al. 2020), a coalescent-based simulation tool that can generate genetic data for large sample sizes.

For the simple model, we used msprime to simulate the population history in Fig. 3, with $\tau_{A}=\tau_{C}=0.25$, and the effective population size is 2,000 for both populations. See the code in Supplementary Section S6.1 in the supplementary material (van Waaij et al. 2025). We use the anchor population to calculate the frequencies of the alleles, and we randomly pick n=1,2,..,8 individuals from the forensic population and use them to simulate reads with constant coverage 100. For clarity, the forensic population refers to the population from which the sampled individuals originate. We used downsampling to get lower coverages. We have set the error rate equal to 3‰. We have a sequence of length $10^{7}$ and 155,776 SNPs, which have frequencies strictly between 0.0 and 1.0 in the anchor population. The results are found in Section “Simple 2-pop simulations”

We then sought to simulate a more realistic demographic scenario with multiple populations. For this simulation, input demographic data was provided in the Demes format (Gower et al. 2022). Demes offers a way to describe demographic models involving multiple interacting populations, migration events, and growth rates via a YAML-based format. To specify the demographic model, we used a predefined model provided by the Demes specification, accessible via their official website (Popsim Consortium 2022). In particular, the starting point for the model we employed is the Gutenkunst out-of-Africa (OOA) model that describes the demographic history of African, European, and Asian populations (Gutenkunst et al. 2009). This model was then adapted to include the demographic history of Indigenous peoples of the Americas (Lindo et al. 2016).

In our model, we implemented population splits occurring 75 years (3 generations) ago. At this time, each of the four populations were duplicated, creating an identical copy of itself, and all of these new populations were isolated from their original counterparts. These new, isolated populations were referred to as “splits.” Each split maintained the same effective population size as the original population at the moment of the split. With these models finalized, simulations were performed, leading to the generation of VCF files. Details about the demographic model as well as the results are found in the Results in Section “Simulated multiple populations.” The source code can be found in Supplementary Section S6.2 of the supplementary material. The results are found in Section “Simulated multiple populations.”

#### Synthetic empirical data

We combine whole-genome sequencing files to simulate a real-world situation. We call this a synthetic empirical data set. The origin and number of contributing individuals are known. We mixed several individuals with data from the 1000 Genomes Project (2015 Genomes Project Consortium et al. 2015), to create a realistic environmental DNA sample. Briefly, we took BAM files from 8 individuals pertaining to the the Iberian population (IBS). We downsampled to create files of relatively equal coverage and merged the files in equal proportions to create a realistic synthetic empirical BAM file. We simulated 1, 2, 4 and 8 individuals. The final coverage for all BAM files ranged from 10X for the BAM file with 1 individual, 7X for 2, 12X for 4 and 24X for the BAM with 8 individuals (estimated using mosdepth Pedersen and Quinlan 2018), which were further downsampled to test the robustness of the method. The results are found in Section “Synthetic empirical data.”

### General idea of our method

In this paper, we rely on the model described by Schraiber (2018), who calculates probabilities of having a total of *k* derived alleles at a certain locus among *n* diploid individuals and with given drift times. We use this by putting an additional prior on *n* and the drift parameters, to estimate *n* and the drift parameters simultaneously, and provide credible intervals as well.

To provide an insight as to how we can estimate the number of individuals, *n*, we will show that the distributions for different *n* differ from each other. For a certain locus, assume the derived allele in the population the sample pertains to has frequency x∈[0,1]. Under the Hardy-Weinberg equilibrium, the total number of derived alleles, *k*, among the *n* diploid individuals is binomially distributed with parameters 2n and *x*. Given k∈{0,…,2n} derived alleles, the number of derived reads is binomially distributed with parameters *R* and k/(2n), where *R* is the coverage. Thus, the derived read count follows a mixture of 2n+1 binomials. Note that we do not require knowing which read comes from which individual.

**Fig. 2.**
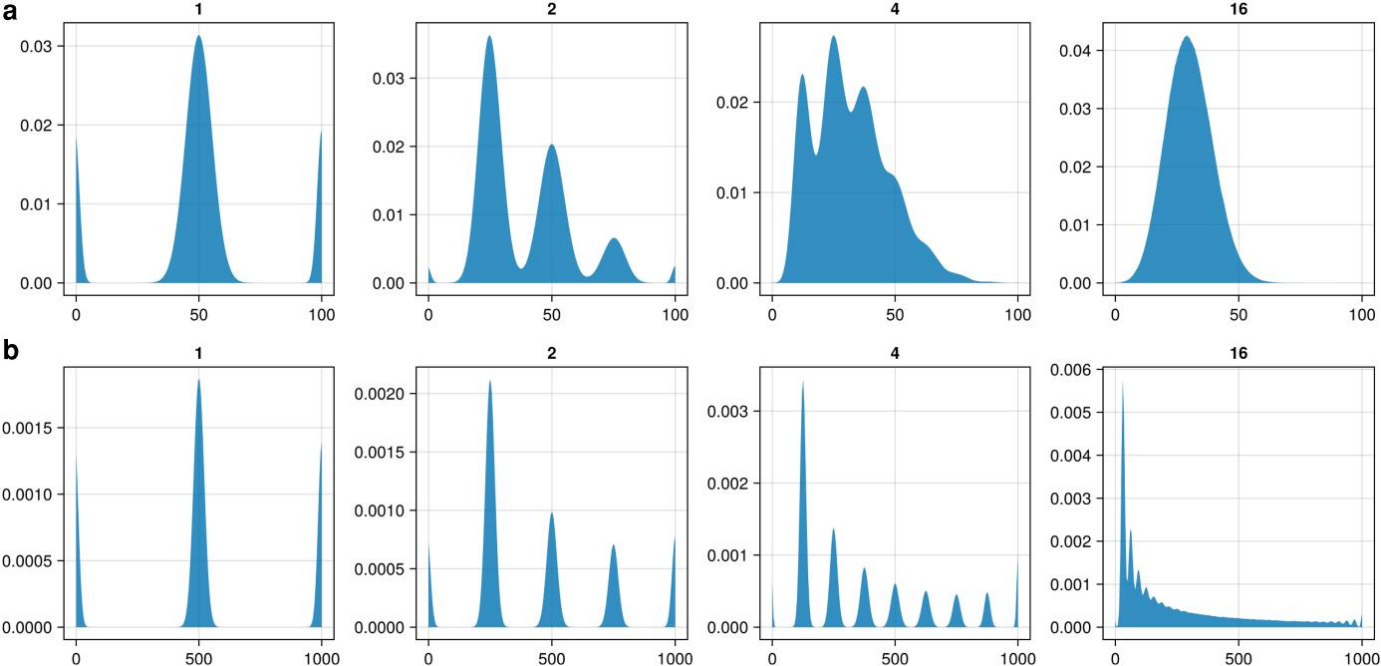
a) Histograms simulating the read distributions for an allele frequency of 0.3 and 10M replicates. The read distribution from binom(100,k/(2n)), where *k* in turn is simulated from binom(2n,0.3), for 1, 2, 4, and 16 individuals. b) Same as above but for all sites in the genomes where the derived read frequencies are from a site frequency spectrum. For a single individual, the distribution is a binomial centered around 0.5 however as the number of individuals increases, this distribution converges to the site frequency spectrum.

For large *n*, the distribution of the reads converges more and more to a binomial distribution with parameters *R* and *x* (Supplementary Lemma S4 and Section S1 in the supplementary material). As *n* is small, the different modes are easily distinguishable, which makes it easy to estimate *n*. For larger *n*, it is harder. To illustrate this principle, we simulate *k* derived alleles with frequency 0.3, and given *k* derived alleles, we simulate derived reads with probability k/(2n) and coverage 100. The densities for the different *n*, based on 10M simulation are shown in the top row of Fig. 2. We do the same where the frequency is distributed with the site frequency spectrum (instead of being fixed at 0.3). This gives the bottom row of Fig. 2. For larger *n*, the distributions become less distinguishable. Consequently, it is also harder to estimate *n*, however, if we are only interested in estimating the drift parameters, then for larger *n*, the exact value becomes less important.

**Fig. 3.**
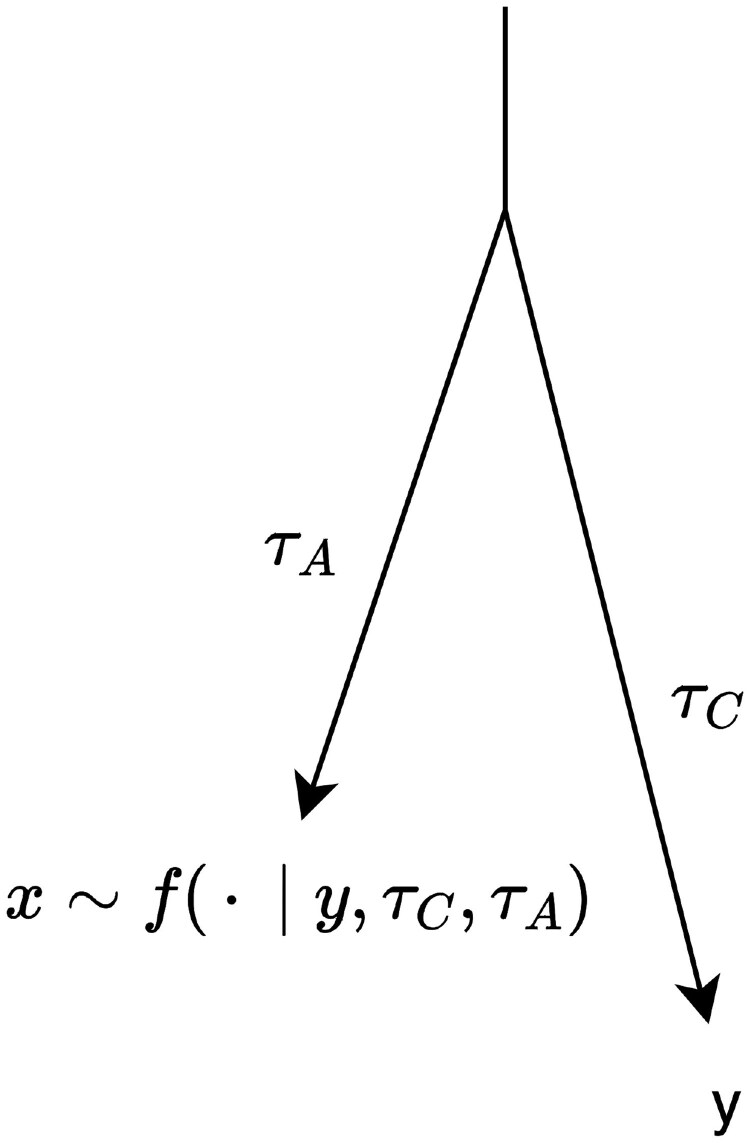
$\tau_{C}$ and $\tau_{A}$, model parameters that represent the drift times of the anchor population and sample population, respectively. We seek *x*, the derived allele frequency in the sample population, with distribution *f* which itself depends on $\tau_{C}$ and $\tau_{A}$ and *y*.

### The demographic model

In this section, we describe our statistical method for correctly estimating the number of individuals, the drift parameters, and the error rate.

Let us consider a fixed locus with a derived allele frequency y∈(0,1) in a related population for which we have high-resolution allele frequencies. We call this population the anchor population. We assume that the forensic and anchor populations have a common ancestor. Since the common ancestor $t_{C}$ generations have passed until the arrival of the anchor population, and $t_{A}$ generations have passed until the forensic population, from which we have our eDNA or forensic sample. Let $\tau_{C}=\frac{t_{C}}{2N_{C}}$ and $\tau_{A}=\frac{t_{A}}{2N_{A}}$ be the corresponding drift times, where $N_{C}$ is the effective population size of the anchor population and $N_{A}$ is the effective population size of the forensic population.

We assume that the frequency of the derived allele in the forensic population has a distribution *f*, which depends on $\tau_{C},\tau_{A}$ and *y*. We denote this dependency by $f(\;\cdot \;)=f(\;\cdot \;|y,\tau_{A},\tau_{C})$. We consider *f* later.

So, at this locus, in the forensic population, the frequency *x* of the derived allele is a realization of *f*. Given *x*, under the Hardy-Weinberg equilibrium, the 2n alleles of the *n* individuals have each, independently, a probability *x* of being derived, so the total number of derived alleles *k* is binomially distributed with parameters 2n and *x*, so the probability of *k* derived alleles is


(2nk)xk(1−x)2n−k.

So with *k* derived alleles, a read has a chance of $\frac{k}{2n}$ of being derived. However, through deamination or read errors, a derived read or allele might become (apparently) ancestral or vice versa, so the probability of a derived read with error rate ε becomes


$$(1-\varepsilon )\frac{k}{2n}+\varepsilon \frac{2n-k}{2n}.$$

So the probability of *d* derived reads, conditioned on *k* derived alleles and error rate ε, with coverage *R* is binomially distributed with parameters *R* and $(1-\varepsilon )\frac{k}{2n}+\varepsilon \frac{2n-k}{2n}$. In Supplementary Section S3.1 in the supplementary material (van Waaij et al. 2025), we argue that a separate estimation of the deamination and read error, under this model, is impossible. In addition, to avoid identifiability issues, we assume ε∈[0,1/2) (see Supplementary Section S3.2 in the supplementary material  van Waaij et al. 2025). So, in a scheme, we have


$$\begin{array}{rl} x∼ & f\\ k|x∼ & \text{binom}(2n,x)\\ d|k,\varepsilon ∼ & \text{binom}\left(R,(1-\varepsilon )\frac{k}{2n}+\varepsilon \frac{2n-k}{2n}\right). \end{array}$$

Schraiber (2018) calculates the formula for the integral $\int_{0}^{1}x^{k}(1-x{)}^{2n-k}f(x)dx$, for all *n* and *k*. He found that the probability of *k* derived alleles out of 2n, given frequency *y* in the anchor population and drift parameters $\tau_{C}$ and $\tau_{A}$, to be


P(k∣n,y,τC,τA)=(2nk)p(k,n,y,τC,τA),

where


$$p(k,n,y,\tau_{C},\tau_{A})={\left(e^{Q\tau_{A}}e^{Q^{↓}\tau_{C}}h(n,y)\right)}_{k+1},$$

and h(n,y) is a real vector of length 2n+1, defined as


$$h(n,y)=\left(\begin{array}{c} (1-y{)}^{2n}\\ y(1-y{)}^{2n-1}\\ \vdots \\ y^{2n} \end{array}\right).$$

and *Q* and $Q^{↓}$ are (2n+1)×(2n+1) tri-diagonal matrices defined (for k,ℓ∈{0,…,2n}) by


$$Q_{k+1,\ell +1}=\left\{ \begin{array}{cc} \frac{1}{2}k(k-1)\; & \text{if}\;\ell =k-1,\\ -k(2n-k) & \text{if}\;\ell =k,\\ \frac{1}{2}(2n-k)(2n-k-1)\; & \text{if}\;\ell =k+1,\\ 0 & \text{otherwise}, \end{array}\right.$$

and


$$Q_{k+1,\ell +1}^{↓}=\left\{ \begin{array}{cc} \frac{1}{2}k(k-1)\; & \text{if}\;\ell =k-1,\\ -k(2n-k+1) & \text{if}\;\ell =k,\\ \frac{1}{2}(2n-k+1)(2n-k)\; & \text{if}\;\ell =k+1,\\ 0 & \text{otherwise.} \end{array}\right.$$

(Note that in mathematics, matrices and vectors traditionally start with index 1.) So for a given locus, the probability of *d* derived reads out of *R*, with *n* individuals, frequency *y* in the anchor population, $\tau_{C}$ and $\tau_{A}$ and error rate ε, is


P(d∣R,n,y,τC,τA)=∑k=02n(Rd)((1−ε)k2n+ε2n−k2n)d(1−(1−ε)k2n−ε2n−k2n)R−dP(k∣n,y,τC,τA).

Schraiber (2018) argues in the appendix of his article that loci with exactly 1X coverage do not contribute information about $\tau_{A}$. In addition, we argue in Supplementary Section S3.3 that loci with 1X coverage also do not provide information about the number of individuals. Loci with coverage one only help to estimate $\tau_{C}$ and the error rate ε. These facts limit estimating $\tau_{A}$ and *n* with low coverage data, under this model.

### The metropolis sampler

As we pointed out at the start of this section, correct estimation of the number of individuals is crucial, as conditioning on different *n* leads to different distributions of the reads, even with the same frequency.

Early tests showed that incorrect estimation of *n* can lead to erroneous estimates of the drift parameters. When we set a uniform distribution on the drift parameters, the posterior concentrates around significantly different values of $\tau_{C}$ and $\tau_{A}$ for different *n*; this makes the correct estimation of *n* essential and simultaneously very challenging. Suppose at step m−1 we are at position $\left(n_{m-1},\tau_{C,m-1},\tau_{A,m-1}\right)$, then if we were to propose a different $n_{\;prop}$ with proposed $\tau_{C}$ and $\tau_{A}$ values close to $\tau_{C,m-1}$ and $\tau_{A,m-1}$ (as is, with high probability, done in random walk MCMC), then this proposal is almost certainly rejected, because the posterior conditioned on $n_{\;prop}$ individuals concentrates around other $\tau_{C}$ and $\tau_{A}$ values. However, simulations show that conditioned on *n*, the posterior is unimodal. In conclusion, a naive symmetric random-walk Metropolis sampler leads to samples that do not mix and often estimate the wrong number of individuals. Typically, different chains get stuck at different *n*. To remedy this, we crafted an new MCMC sampler that can handle this mixture model.

The idea is as follows. For every *n*, we run a separate Metropolis sampler of the posterior distribution conditioned on having *n* individuals. To make this practical, we should assume that the number of individuals is bounded by some known $n_{\max}$. Then we have an extra sampler $(n_{m}{)}_{m\geq 1}$ for the posterior distribution of the number of individuals, where at step *m*, the proposal is accepted depending on the conditional drift values in the $n_{\max}$ other chains at step *m*. Finally, from these $n_{\max}+1$ chains we construct one unconditioned posterior sample, by taking $n_{m}$ and the drift parameters and error estimate corresponding to conditioning on $n_{m}$. So, if the conditioned chains are $(\tau_{C,1,m},\tau_{A,1,m}{)}_{m\geq 1}$, …, $(\tau_{C,n_{\max},m},\tau_{A,n_{\max},m}{)}_{m\geq 1}$, then we take $(n_{m},\tau_{C,n_{m},m},\tau_{A,n_{m},m}{)}_{m\geq 1}$ as the sample from our unconditioned posterior. The theoretical justification can be found in Supplementary Theorem S5 in the supplementary material (van Waaij et al. 2025). The sampler is described in detail in Supplementary Section S2.1 in the supplementary material (van Waaij et al. 2025).

#### Software implementation

We implemented the model and the MCMC sampler in CHARON in the Julia programming language (Bezanson et al. 2017). Installation, test data and usage can be found in the GitHub repository: https://github.com/Jan-van-Waaij/Charon. BAM files can be easily transformed in our native format using glactools (Renaud 2018) and scripts provided with the software.

It is possible to sample several chains in parallel. For this, one has to set the --threads flag of Julia (which requires Julia 1.5 or higher; for older versions of Julia, one has to set the JULIA_NUM_THREADS environment variable). For example, Julia could start with julia --threads=4 to run four chains in parallel.

For efficient calculation of the matrix exponent in the likelihood, we make use of the fact that one can factorize *Q* and $Q^{↓}$ as $M=PDP^{-1}$, where *P* is an invertible matrix and $D=\text{diag}(d_{1},\ldots ,d_{n})$ is a diagonal matrix with entries $d_{1},\ldots ,d_{2n+1}$. Then $e^{\tau M}=PEP^{-1}$, where $E=\text{diag}(e^{\tau d_{1}},\ldots ,e^{\tau d_{2n+1}})$ is a diagonal matrix with entries $e^{\tau d_{1}},\ldots ,e^{\tau d_{2n+1}}$.

## Results

### Simulations

To show the effectiveness of our method, we first tested it with simulations. We want to show that our method (i) can estimate the number of individuals accurately. (ii) can estimate the population parameters and the error rate accurately. (iii) Pinpoint the most likely population that contributed to the sample. To achieve goals (i) and (ii), we first simulate a simple population model with known drift parameters, see Section “Simple 2-pop simulations.” For goal (iii), we simulate a population history, see Section “Simulated multiple populations.” For goals (i) and (iii), we also constructed synthetic empirical data, see Section “Synthetic empirical data.”

#### Simple 2-pop simulations

In order to show the effectiveness of our method to estimate the correct number of individuals and the population parameters, we simulated a two-population history where the parameters are exactly known (previously described in Section “Simulated data”). Using the simulations, we subsample the reads to simulate lower coverage by using sampling without replacement. Thus the distribution of the reads remains the same (see Supplementary Lemma S2 in the supplementary material).

We apply our MCMC sampler and obtain a chain of 100,000 samples. We use the last 50,000, which are then thinned by using only every 10th element of the sample, resulting in 5,000 draws from the posterior.

We calculate 95% credible intervals, by taking the 2.5% and 97.5% quantiles of the posterior sample. The results are shown in Supplementary Figs. S2 to S5 in the supplementary material (van Waaij et al. 2025). Each plot has credible intervals for coverage 1, 2,…100. The black dot is the posterior median, and the horizontal coloured lines are the 95% credible intervals. The vertical blue line indicates the true simulated value.

**Fig. 4.**
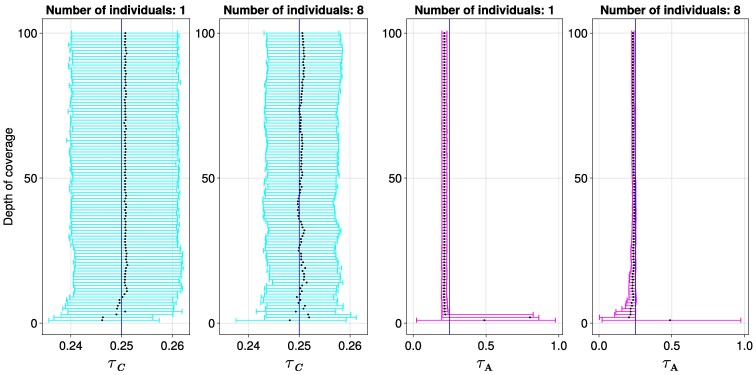
Credible intervals for $\tau_{C}$ (cyan) and $\tau_{A}$ (magenta), for 1 and 8 individuals, coverages 1,…,100. The blue line indicates the true simulated value. The black dot indicates the median.

For the estimation of $\tau_{C}$, our estimates seem robust down to 1X. The intervals tend to become slightly smaller for a larger number of individuals. For illustration, we only show here in Fig. 4 the plots for one and eight individuals. The other plots are shown in the supplementary material Fig. S2.

For the estimation of $\tau_{A}$ the credible intervals get narrower as the depth of coverage increases. The number of individuals does not seem to affect the estimate. For illustration, we only show in Fig. 4 the case with one and eight individuals. In the supplement, all plots are shown, see Supplementary Fig. S3.

**Fig. 5.**
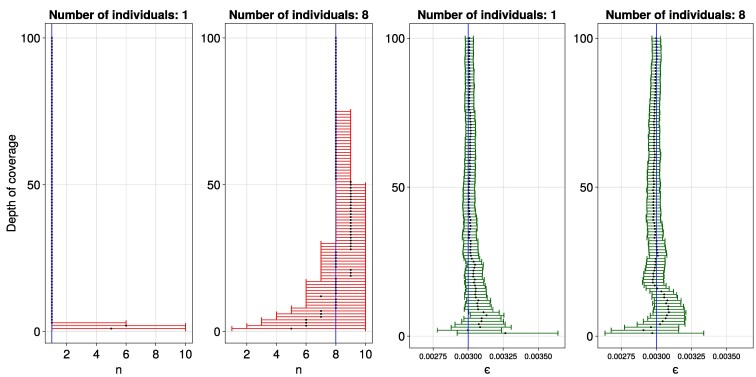
Credible intervals for *n* (red) and ε (green), for 1 and 8 individuals, coverages 1,…,100. The blue line indicates the true simulated value. The black dot indicates the median. For higher depth of coverage, the 95% credible intervals of *n* consist of only one point.

**Fig. 6.**
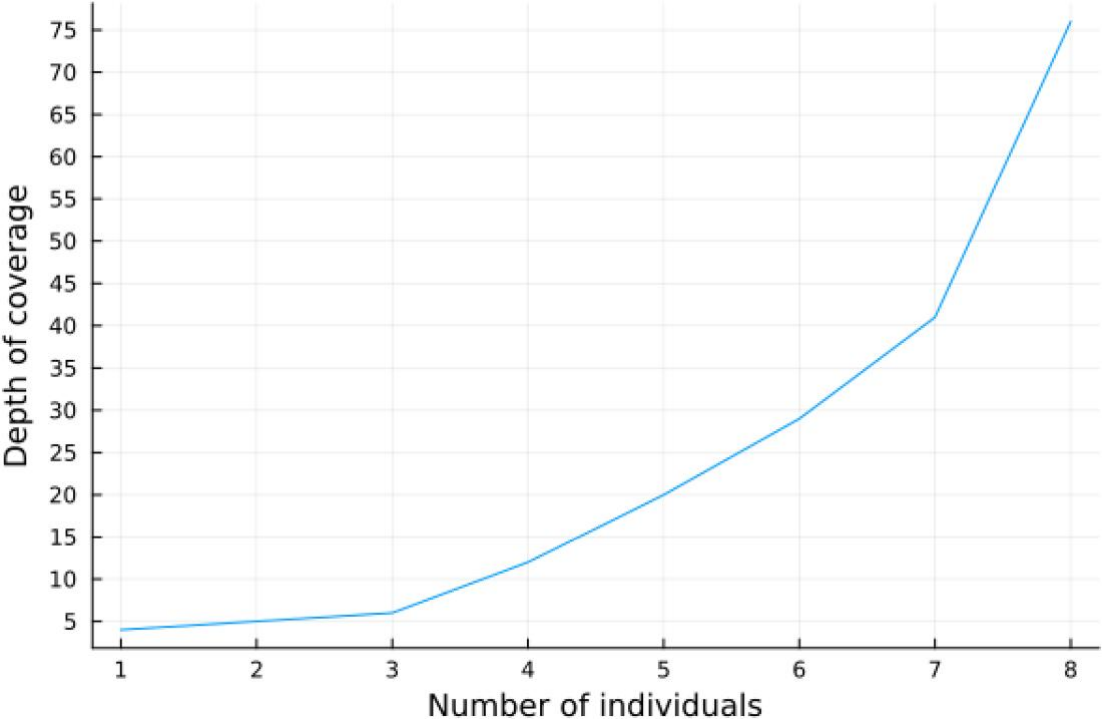
The minimum required coverage so that the posterior puts at least 95% mass on the true value of *n*.

The estimation of the number of individuals *n* converges to the right answer as coverage increases. For a small number of individuals (e.g. 1,2), we need depth of coverage of 2X–3X, for a larger number of individuals (e.g. 8), the uncertainty is higher and we require more than 50X for a perfect point estimate but roughly 10X to get a confidence interval of plus or minus 2 individuals. For the most difficult case, 8 individuals with 1X coverage, our estimate of the number of individuals is between 1 and 10, meaning that we cannot predict with certainty the number of individuals. The credible interval gets narrower and narrower around the correct value as coverage increases. For illustration, we only show here in Fig. 5 the plots for one and eight individuals. In the supplementary material Fig. S4, all plots are shown. Note that the credible intervals might be asymmetric and skewed because the posterior puts almost all mass on one value, and a little bit on either the smaller or higher values, and almost no mass on the remaining values, resulting in asymmetrical, skewed intervals. In Fig. 6 we show the minimum coverage needed so that the posterior puts at least 95% posterior mass on the true number of individuals.

For the estimation of the error rate ε, there is no notable difference between the number of individuals. The estimates become narrower as the depth of coverage increases and converge around the correct value. For illustration, we only show here in Fig. 5 the plots for one and eight individuals. In the supplementary material Fig. S5 all plots are shown.

#### Simulated multiple populations

Here we will show that our method is able to pinpoint the right population. We simulated a human population history with Demes and msprime as described in Section “Simulated data” and show that our method can accurately pinpoint the population and also estimate the correct number of individuals.

**Fig. 7.**
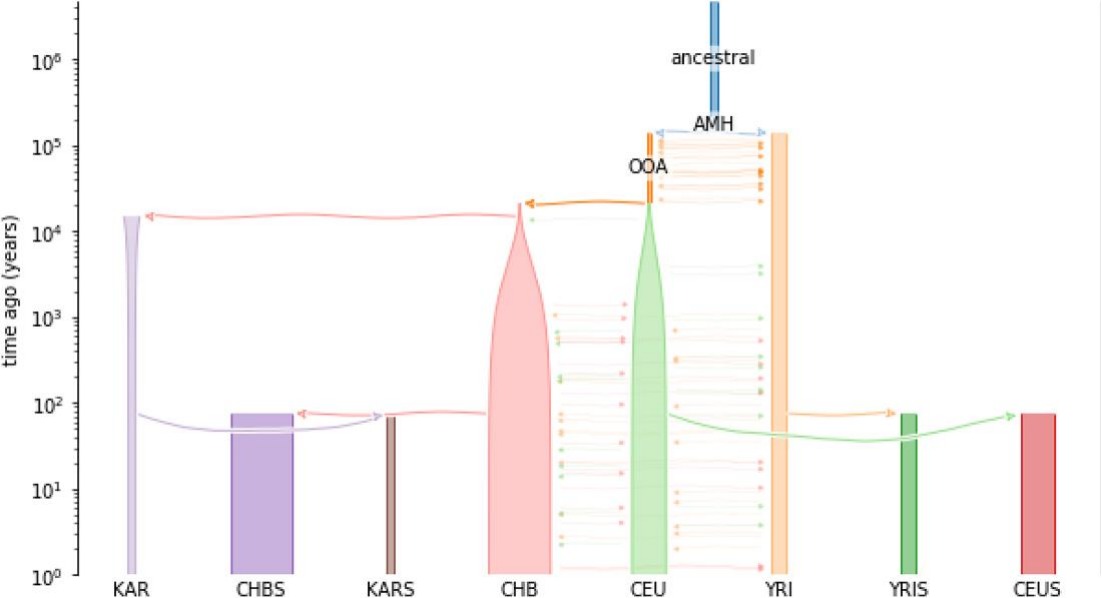
From the ancestral human population in Africa, we have different migrations and splits in new parts of the world. This is visualized here as the YRI staying in Africa, OOA being the emigration leading to the CEU, CHB and KAR populations. For this study, each population also has a split population used to generate the individual samples. We used a split of 75 years (3 generations) ago for each sample. This is done to simulate the passage of time between the split from the population in the sample versus the anchor population. For the abbreviations see Table 1.

The human population history being simulated follows an out-of-Africa split, followed by a subsequent one in Eurasia and the settling of the Americas (see Fig. 7). The abbreviations are in Table 1.

**Table 1.** Abbreviations for the different populations.

**AMH**	Anatomically modern humans
**OOA**	Bottleneck out-of-Africa population
**YRI**	Yoruba in Ibadan, Nigeria
**YRIS**	Yoruba in Ibadan, Nigeria - Split
**CEU**	Utah residents with northern and western European ancestry
**CEUS**	Utah residents with northern and western European ancestry - split
**CHB**	Han Chinese in Beijing, China
**CHBS**	Han Chinese in Beijing, China - split
**KAR**	Karitiana native Brazilians
**KARS**	Karitiana native Brazilians - split

We took the populations KAR, CHB, CEU, and YRI as anchor populations, and we simulated eDNA from the populations CHBS, KARS, YRIS and CEUS, which split from their respective populations 75 years (3 generations) ago. We estimated the confidence interval for each of the combinations of populations with coverage 5, 10,…, 100. For the number of individuals, we took 1, 2,…, 8. The simulated error rate is 0.003. The plots can be found in Supplementary Figs. S10 to S73 in the supplementary material (van Waaij et al. 2025).

**Fig. 8.**
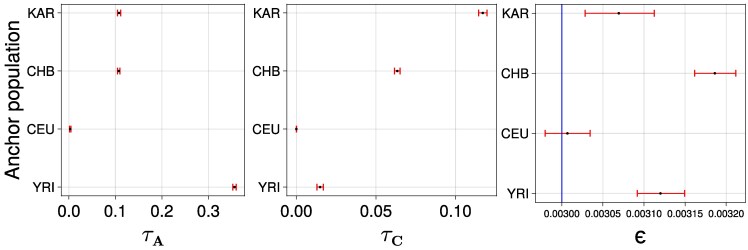
Credible intervals for the drift in the branch leading to the sample $\tau_{A}$ (left) and drift in the branch of the anchor population $\tau_{C}$ (middle), with n=3 individuals and a coverage of 20X. To the right is the credible interval for ε. The blue line is the simulated true error level. For the abbreviations we refer to Table 1.

The number of individuals and the error rate are correctly estimated. The credible intervals for the number of individuals can be found in Supplementary Figs. S13, S17, S21, S25, S29, S33, S37, S41, S45, S49, S53, S57, S61, S65, S69 and S73 in the supplementary material (van Waaij et al. 2025) and the plots for the credible intervals of the error rate can be found in Supplementary Figs. S12, S16, S20, S24, S28, S32, S36, S40, S44, S48, S52, S56, S60, S64, S68 and S72 in the supplementary material (van Waaij et al. 2025). In the data with three individuals and coverage 20X, the posterior correctly places more than 95% of its mass on n=3. The credible intervals for the estimation error are given in Fig. 8. We see from Fig. 8 that our method accurately infers the short distance both in terms of $\tau_{A}$ and $\tau_{C}$ between CEU and CEUS. (For the abbreviations we refer to Table 1.) The plots for the other sample populations can be found in Supplementary Figs. S6 to S9. For $\tau_{A}$, we are mostly dependent on the split time hence the higher value for YRI. For $\tau_{C}$, we are both dependent on the split time and effective population size in the anchor population. Due to the lower effective population size in the KAR, we see the highest value for this parameter followed by CHB and YRI. This indicates that we can use our method to determine the population that is the genetically closest one to the eDNA sample.

### Synthetic empirical data

Using the synthetic empirical data described in Section “Synthetic empirical data,” we sought to determine if our method could identify the number of individuals as well as the source population. For the latter, we use different statistics to determine the shortest distance to the sediment population from several anchor populations, that is, the median of $\tau_{C}$, $\tau_{A}$, $\max\left\{\tau_{A},\tau_{C}\right\}$ and $\tau_{A}+\tau_{C}$.

**Fig. 9.**
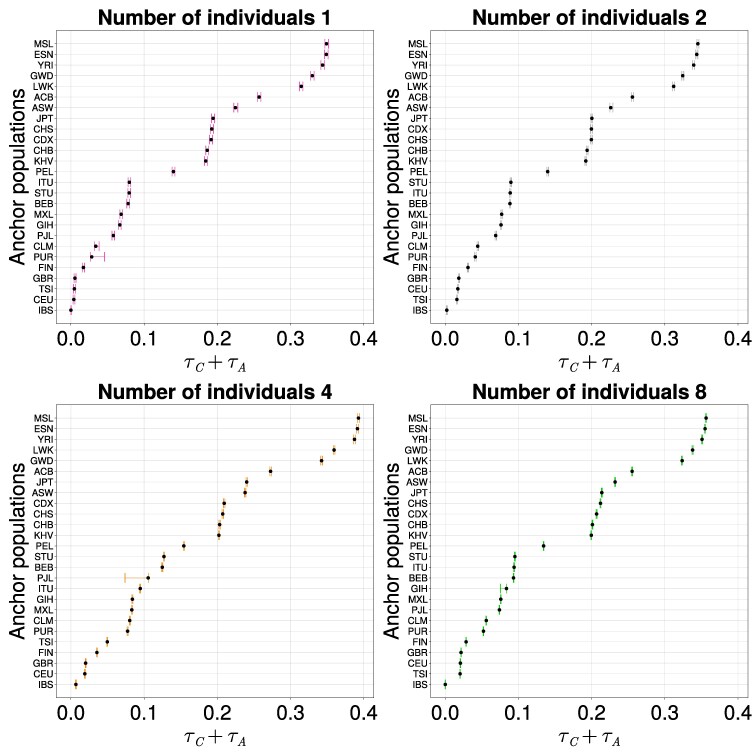
Populations ordered by $\tau_{C}+\tau_{A}$ for the samples simulated with IBS as source population, respectively, along with 95% credible intervals. The black dot indicates the posterior median. For the abbreviations we refer to Table 1.

The results of the different population drift statistics are in Supplementary Fig. S75 in the supplementary material (van Waaij et al. 2025). Here, in Fig. 9, we only show the results for $\tau_{C}+\tau_{A}$. In general, we see that when considering $\tau_{C}+\tau_{A}$ hence the total drift between the sample and anchor population, the population with the lowest value is IBS followed by either Tuscans (TSI) or Northern European (CEU) whereas the highest values are for the Mende (MSL) and Esan (ESN). These results are expected due to the demographic history. Regardless of the number of individuals, IBS was always the population with the lowest drift values.

**Fig. 10.**
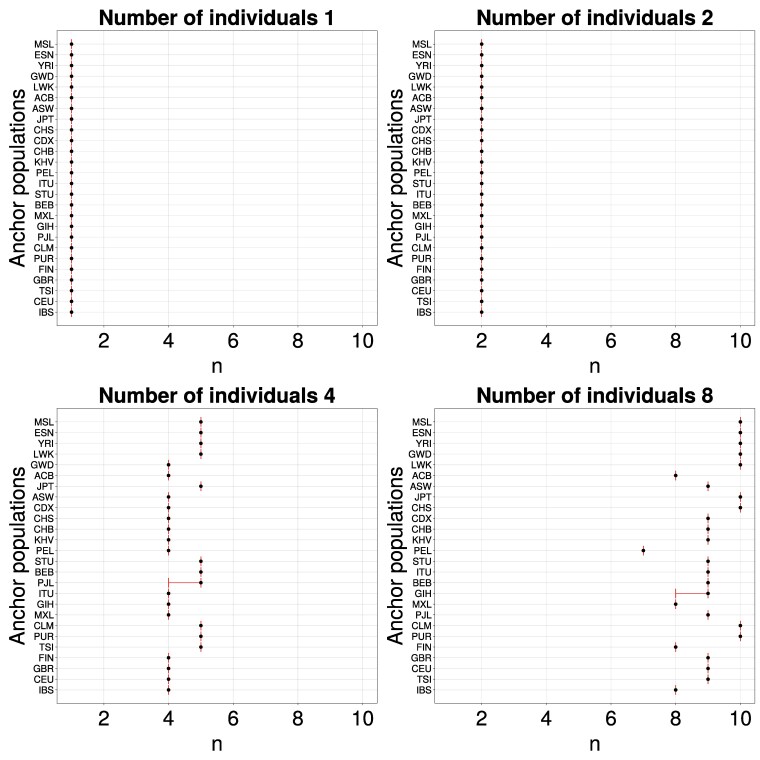
Credible intervals for the number of individuals, except for a few cases, the posterior puts almost all its mass in one point. The black dot indicates the posterior median. For the abbreviations we refer to Table 1.

We also estimate the number of individuals (see Fig. 10). We see that our estimates are correct for the number of individuals for n=1,2 regardless of the anchor population. At n=4, our estimate is mostly correct, especially for populations with lower drift values (e.g. IBS, TSI, CEU). These estimates are off by one for the population with higher drift values (e.g. ESN, MSL), probably due to the increasing lack of allele frequency resolution at high drift. At n=8, we often overestimate by 1 or 2 for most populations but using IBS as anchor gives us the correct estimate.

Thus, for small values of *n*, our method accurately recovers the number of individuals. Moreover, it consistently identifies the correct population structure, with drift patterns aligning with known demographic histories. The credible intervals for the error rates ε are in Supplementary Fig. S74 in the supplementary material (van Waaij et al. 2025).

## Discussion

In this study, we introduced CHARON, a novel statistical method designed to address two significant challenges in environmental and forensic DNA analyses: accurately estimating the number of contributing diploid individuals and inferring genetic drift times between a sampled population and known reference populations. Using allele frequency distributions from known populations, CHARON provides a statistical framework for jointly estimating these parameters.

Our simulation studies demonstrated that CHARON accurately estimates the number of individuals contributing to DNA mixtures at mid-coverage (above ∼3X for a 1–2 of individuals, above 8-10X for 8 individuals), distinguishing up to eight diploid individuals. Beyond this, CHARON runs into numerical and statistical issues. Despite this, as the coverage increases, the credible intervals for estimates of drift parameters ($\tau_{C}$ and $\tau_{A}$), the error rate, and the number of individuals narrow, confirming the robustness of the model.

When applied to simulated population data, CHARON effectively pinpointed the correct population of origin. It accurately inferred smaller drift times between closely related populations (e.g. CEU and its split CEUS, for the abbreviations of the populations, see Table 1) while providing wider drift intervals for more genetically distant populations. Using synthetic empirical data constructed from the 1000 Genomes Project, CHARON correctly identified the population of origin (IBS) and accurately estimated the number of individuals across different simulated scenarios. The reasonable depth of coverage inherent to these synthetic mixtures (8X-10X), showed that our method is applicable to empirical human eDNA and forensic contexts. It is plausible that even ancient sediments will yield enough human DNA to allow quantification of the population of origin and estimate the number of individuals.

However, CHARON assumes equal contributions from all individuals and a homogeneous genetic background within the sampled population. These assumptions, while practical, could limit applicability in scenarios involving highly skewed individual contributions or heterogeneous samples. Future work might explore extensions of CHARON to account for unequal DNA contributions or mixtures from genetically diverse populations.

## Supplementary Material

jkag099_Supplementary_Data

## Data Availability

We used msprime (Kelleher et al. 2020) to generate the data in Section “Simulated data.” The data are available on ?. The data in Section “Synthetic empirical data” is generated from data from the 1000 Genomes Project (2015 Genomes Project Consortium et al. 2015). The software is available on Github (van Waaij 2025). There is complementary material to this article with additional figures and mathematical theory (van Waaij et al., 2025). Supplemental material available at G3 online.
